# Peiminine Suppresses RANKL-Induced Osteoclastogenesis by Inhibiting the NFATc1, ERK, and NF-κB Signaling Pathways

**DOI:** 10.3389/fendo.2021.736863

**Published:** 2021-09-24

**Authors:** Mengbo Zhu, Wenbin Xu, Jiuzhou Jiang, Yining Wang, Yanjing Guo, Ruijia Yang, Yaqiong Chang, Bin Zhao, Zhenyu Wang, Jianfeng Zhang, Te Wang, Liqin Shangguan, Shaowei Wang

**Affiliations:** ^1^ Department of Orthopedic, Second Hospital of Shanxi Medical University, Taiyuan, China; ^2^ Department of Orthopedic Surgery, Sir Run Run Shaw Hospital, Medical College of Zhejiang University, Hangzhou, China; ^3^ Department of Orthopaedics, The Second Affiliated Hospital and Yuying Children’s Hospital of Wenzhou Medical University, Wenzhou, China; ^4^ Department of Biochemistry, Basic Medical College, Shanxi Medical University, Taiyuan, China

**Keywords:** peiminine, osteoclast, NFATc1, NF-κB, osteoporosis

## Abstract

Osteoclasts (OCs) play an important role in osteoporosis, a disease that is mainly characterized by bone loss. In our research, we aimed to identify novel approach for regulating osteoclastogenesis and thereby treating osteoporosis. Previous studies have set a precedent for screening traditional Chinese herbal extracts for effective inhibitors. Peiminine is an alkaloid extracted from the bulb of *Fritillaria thunbergii Miq* that reportedly has anticancer and anti-inflammatory effects. Thus, the potential inhibitory effect of peiminine on OC differentiation was investigated *via* a series of experiments. According to the results, peiminine downregulated the levels of specific genes and proteins *in vitro* and consequently suppressed OC differentiation and function. Based on these findings, we further investigated the underlying molecular mechanisms and identified the NF-κB and ERK1/2 signaling pathways as potential targets of peiminine. *In vivo*, peiminine alleviated bone loss in an ovariectomized mouse model.

## Introduction

Osteoporosis, common in elderly individuals, especially women, is characterized mainly by microarchitectural degeneration of the trabeculae and intrinsic bone tissues as well as pathological bone remodeling (1). Due to these pathological changes, which are often described as bone loss, patients suffering from osteoporosis are at an enhanced risk for osteoporosis-related fractures (2). A critical cause of bone loss is the imbalance between bone formation and osteoclast (OC)-related bone resorption (3). Estrogen replacement therapy (ERT) is considered to be effective for menopausal osteoporosis but increases the risk of endometrial cancer, breast cancer, and asthma (4, 5), and bisphosphonates are the mainstays for the in-clinical treatment of osteoporosis. However, bisphosphonates exhibit nephrotoxicity, hepatic toxicity, and alimentary canal toxicity (6). Thus, we expect to explore safer strategies for regulating the differentiation of OCs to treat osteoporosis. Natural compounds with a broad spectrum of biological activity and limited side-effects have become the main targets of our research. There have been many studies on the treatment of osteoporosis using natural compounds (7–9) that have provided us with a reliable theoretical and technological basis for our exploration into the field.

**Figure 1:**
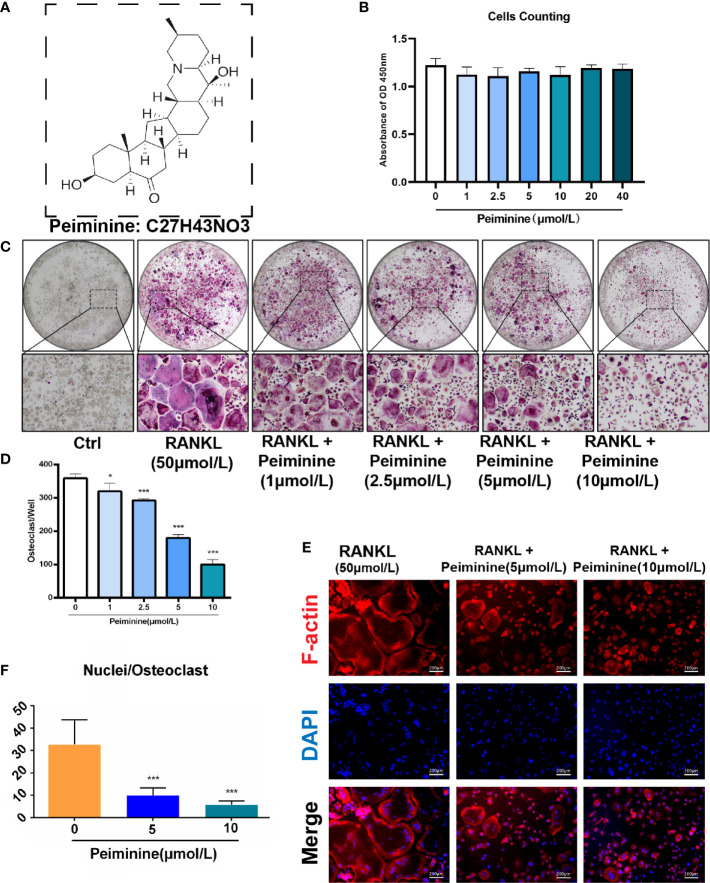
Peiminine dose-dependently inhibits osteoclastogenesis induced by RANKL and is not cytotoxic *in vitro*. **(A)** The molecular structure of peiminine. **(B)** Evaluation of the cytotoxicity of peiminine by the CCK-8 assay. n= 5, **(C)** TRAP staining of OCs treated with peiminine at every concentration. **(D)** Quantitative analysis of the TRAP-positive cells with more than 3 nuclei in each well of a 96-well plate. n= 5, *P < 0.05, ***P < 0.001. **(E)** Fluorescence staining images of OCs treated without or with peiminine at different concentrations (5 µmol/L and 10 µmol/L). The scale bar is 200 µm. **(F)** Analysis of the average number of OC nuclei (dots with blue fluorescence indicated by DAPI staining). n= 5, ***P < 0.001.

Peiminine is an alkaloid extracted from the bulb of *Fritillaria thunbergii Miq*, a traditional Chinese medicinal herb (for the chemical structure of peiminine, see **Figure 1A**) (10). A study performed in 2018 reported that peiminine exerted a protective effect on lipopolysaccharide-induced mastitis by repressing signaling pathways such as protein kinase B (Akt), nuclear factor-κB (NF-κB), and mitogen-activated protein kinases (MAPKs) (11), and peiminine was shown to similarly protect dopaminergic neurons from neuroinflammation by inhibiting the extracellular-regulated protein kinase (ERK1/2) and NF-κB pathways in another study (12). The process of osteoclastogenesis shares these pathways.

OCs are multinuclear giant cells derived from bone marrow monocytes (BMMs) (13), and the receptor activator of nuclear factor-κB ligand (RANKL)-receptor activator of nuclear factor-κB (RANK)-osteoprotegerin (RANK-RANKL-OPG) signaling pathway is the most influential mechanism known to regulate the process of OC differentiation (3, 13–15). Groping towards this way, NF-κB and ERK pathways are all crucial downstream signaling pathways of RANKL-RANK axis (16–18). Hence, blocking these pathways is probably a promising direction of osteoporosis treatment.

We herein hypothesized that peiminine exerts similar inhibitory effects on osteoclastogenesis. Besides, many previous studies have reported that peiminine has certain anticancer effects (19, 20) and is also effective against acute and chronic inflammatory injury (21–24). In the context of orthopedic diseases, peiminine is known to protect articular cartilage from destructive cytokines (25), but its inhibitory effect on OCs and its effect on bone loss have not yet been reported. Thus, this study aimed to provide a novel and comprehensive solution for combating degenerative and aging-related bone diseases.

In our study, we found for the first time that peiminine suppressed osteoclastogenesis by inhibiting the NF-κB signaling pathway. The expression of OC-related genes and proteins was measured to evaluate the influence of peiminine on OC differentiation and function. Our results indicated the alleviating promise of peiminine for osteolytic bone diseases, and its effect on bone loss alleviation was confirmed by animal experiments *in vivo*. The results obtained from a mouse model of osteoporosis induced by estrogen deficiency indicated the potential of peiminine as an alleviating option for osteoporosis.

## Methods and Materials

### Cell Culture

The indispensable basic cell culture materials were produced by Thermo Fisher Scientific (Carlsbad, CA, USA), including alpha-modified minimal essential medium (α-MEM), penicillin/streptomycin (P/S), and fetal bovine serum (FBS). We extracted BMMs from the tibias and femur marrow of C57BL/6 mice at 6 weeks of age in accordance with the *National Institutes of Health (NIH) Guide for the Care and Use of Laboratory Animals* and the guidelines for *The Laboratory Animal Center of Sir Run Run Shaw Affiliated Hospital of Zhejiang University School of Medicine* (Hangzhou, Zhejiang).

Under sterile conditions, 6-week-old mice were sacrificed, and their tibias and femurs were immediately harvested. The two ends of the bones (osteoepiphysis) were clipped to expose the bone marrow, which was harvested from the diaphysis *via* a 1 ml syringe and placed into medium. After triturating with a pipette, M-CSF was added to the bone marrow cell suspension for BMM sorting. The suspended cells were discarded after 48 h of culture in the presence of M-CSF, and the adherent cells were deemed the BMMs. The medium was comprised of α-MEM, 1% (v/v) P/S, 10% (v/v) FBS, and 50 ng/ml M-CSF.

### Evaluation of Peiminine Efficacy and Toxicity

BMMs were seeded in plates (96-well plate) at a concentration of 8×10^3^ cells per well and cultured in α-MEM (Thermo Fisher Scientific, CA, USA) supplemented with 50 ng/ml M-CSF (R&D Systems, MN, USA) overnight according to the method published by Jin and Chen in 2019 (7, 26). After adherence, the BMMs were stimulated with GST-rRANKL (50 ng/ml) (R&D Systems, MN, USA), and peiminine (Feiyubio, Jiangsu, China, CAS no. 18059-10-4) was added to the cultured cells at varying concentrations (1, 2.5, 5, and 10 μmol/L). The medium was changed every 2 days, and GST-rRANKL and the drug were refreshed every 2 days until the sixth day, when OCs formed. The differentiated OCs were stained with tartrate-resistant acid phosphatase (TRAP) (Sigma-Aldrich Corp., St. Louis, MO, USA) solution after fixation with 2.5% glutaraldehyde (DAMAO chemical reagent factory, Tianjin, China) for 15 min, and the number of cells in each well was then counted under a bright-field optical microscope (Olympus, Tokyo, Japan). Cells with three or more nuclei were considered OCs, and the number of TRAP-positive multinucleated cells was used to indicate the differentiation of OCs.

Additionally, the response of the cells to increasing concentrations of peiminine (1, 2.5, 5, and 10 μmol/L) was assessed. BMMs were treated with varying concentrations of peiminine for 5 days, after which 20 μl of WST-8 solution (Cell Counting Kit-8; Dojindo Laboratories, Kumamoto, Japan) was added to the wells for another 4 h. The absorbance at 450 nm was measured by the enzyme-linked immunosorbent assay (Thermo Fisher Scientific, Waltham, MA, USA).

### Fluorescence Staining

BMMs were seeded in 96-well plates (8×10^3^ cells per well) and cultured in α-MEM containing M-CSF (50 ng/ml) for 24 h; after adherence, the medium was replaced in each well. The control group BMMs were cultured in α-MEM containing M-CSF (50 ng/ml) and GST-rRANKL (50 ng/ml) for 6 days, while the BMMs in the two experimental groups were cultured in α-MEM containing M-CSF (50 ng/ml), GST-rRANKL (50 ng/ml), and peiminine (5 and 10 μmol/L separately) for 6 days (medium refreshed every 2 days). On the seventh day after cell seeding, the cells were fixed with 4% paraformaldehyde, and the membrane permeability was increased with 0.5% Triton X-100 (Sigma-Aldrich Corp., St. Louis, MO, USA).

F-actin filaments were stained with rhodamine phalloidin (Thermo Fisher Scientific, Waltham, MA, USA) after blocking with 5% bovine serum albumin. According to the manufacturer’s instructions, the vial contents were dissolved in 150 µl of anhydrous DMSO to yield a 400× stock solution at a concentration of 2,000 assays/ml, which was equivalent to approximately 66 µM. During the experiment, 0.5 µl of the 400× stock solution was diluted in 200 µl of PBS, and approximately 100 µl was added to each well. After 45 min of staining, the cells were washed with PBS and stained with DAPI, and the results were observed with a fluorescence microscope (CKX53; Olympus, Tokyo, Japan). The numbers of multinucleated cells and nuclei in each multinucleated cell were counted under a fluorescence microscope, and the average number of nuclei in each well was then calculated.

### Bone Resorption Assay *In Vitro*

First, sterile bone slices were placed on the well bottoms of 96-well plates, after which BMMs were seeded onto the bone slices (1×10^4^ cells per well) and cultured in α-MEM containing M-CSF (50 ng/ml) for 24 h; after adherence, the medium in each well was replaced. The negative control group cells were cultured in α-MEM containing M-CSF (50 ng/ml), while those in the positive control group were cultured in α-MEM supplemented with M-CSF (50 ng/ml) and GST-rRANKL (50 ng/ml), and those in the two experimental groups were cultured in α-MEM supplemented with M-CSF (50 ng/ml), GST-rRANKL (50 ng/ml), and peiminine (5 and 10 μmol/L). The cells in all groups were cultured for 14 days, and the medium was changed every 2 days. On the 15th day after cell seeding, the cell cultures were removed, and the bone slices from the different groups were obtained.

To prepare scanning electron microscopy (SEM) samples, adherent cells were removed from the bone slices by washing with 75% alcohol and trypsin to ensure that the bone slices were completely decellularized. Then, these decellularized bone slices were fully dried and covered with conductive carbon powder with a vacuum spray plating device (JEOL Ltd., Japan) prior to SEM. The resorption pits on the dried bone slices were observed by SEM (TM-1000; Hitachi, Tokyo, Japan). ImageJ software was used to measure the gross resorption pit areas as follows: adjust the scanning picture to 8-bit and set an appropriate gray value threshold; select dark recessed areas as the resorption area and deselect all scratch-like recessed areas (scratches were engendered during the process of slice cutting); and calculate the gross resorption pit areas in each well.

### Real-Time Quantitative Polymerase Chain Reaction

BMMs were seeded in 6-well plates (15×10^4^ cells per well) and cultured in α-MEM containing M-CSF (50 ng/ml) for 24 h; after adherence, the medium in each well was changed. Cells in the negative control group were cultured in α-MEM containing M-CSF (50 ng/ml), while those in the positive control group were cultured in α-MEM containing M-CSF (50 ng/ml) and GST-rRANKL (50 ng/ml), and those in the two experimental groups were cultured in α-MEM containing M-CSF (50 ng/ml), GST-rRANKL (50 ng/ml), and peiminine (5 and 10 μmol/L). Cells in all the groups were cultured for 6 days, and the medium was changed every 2 days. On the seventh day after cell seeding, total RNA was extracted from the cells with TRIzol reagent and the Ultrapure RNA Kit (DNase I) (CW Biotech, Beijing, China).

The RNA concentration in each sample was detected by measuring the absorbance at 260 nm with a spectrophotometer (Thermo Fisher Scientific, Waltham, MA, USA). Evo M-MLV RT Premix for qPCR (Accurate Biotechnology, Hunan, China) was used to reverse transcribe the total RNA (500 ng) into single-stranded cDNA.

According to the manufacturer’s protocol, the expression of the target genes was determined with a SYBR polymerase chain reaction Master Mix Kit (Yeasen Co., Shanghai, China) on the ABI QuantStudio 6 Real-Time PCR System (Thermo Fisher Scientific, Waltham, MA, USA). The relative expression levels of the target genes were analyzed by the 2−ΔΔCq method, and the expression level of the mouse glyceraldehyde-3-phosphate dehydrogenase (GAPDH) gene was used as the normalization parameter in our analysis. The primer sequences are listed in **Table 1**.

**Table 1:** Primer sequences used in qRT-PCR.

GENE	FROWARD (5’-3’)	REVERSE (5’-3’)	Tm (℃)
**CTSK**	CCA GTG GGA GCT ATG GAA GA	AAG TGG TTC ATG GCC AGT TC	60
**DC-STAMP**	TTC ATC CAG CAT TTG GGA GT	ACA GAA GAG AGC AGG GCA AC	60
**Acp5**	CAG CAG CCA AGG AGG ACT AC	ACA TAG CCC ACA CCG TTC TC	59
**V-ATPase-d2**	GTG AGA CCT TGG AAG TCC TGA A	GAG AAA TGT GCT CAG GGG CT	60
**Nfatc1**	CAA CGC CCT GAC CAC CGA TAG	GGC TGC CTT CCG TCT CAT AGT	60
**c-Fos**	TTT CAA CGC CGA CTA CGA GG	GCG CAA AAG TCC TGT GTG TT	60
**TNFRSF11**	GAA GAT GCT TTG GTG GGT GT	TCA GTC GGG ATC AGT GTG AG	60
**GAPDH**	ACC CAG AAG ACT GTG GAT GG	CAC ATT GGG GGT AGG AAC AC	60

### Western Blot Analysis

To evaluate the expression of proteins related to the NFATc1 signaling pathway and bone resorption, BMMs were seeded in six-well plates (1.5×10^5^ cells per well) overnight and allowed to adhere. The cells were cultured with or without peiminine (10 μmol/L) in medium containing M-CSF (50 ng/ml) and GST-rRANKL (50 ng/ml), and total proteins were then harvested separately from each well on days 0, 1, 3, and 5. Radioimmunoprecipitation assay (RIPA) lysis buffer (consisting of phosphatase inhibitors, 500 g/ml DNase I, and 100 g/ml PMSF) was used to extract the total protein.

To assess the expression of signaling pathway-related proteins at early time points, BMMs were seeded at a density of 5×10^5^ cells per well in six-well plates and incubated in GST-rRANKL-free medium overnight. The cells were starved for more than 6 h and then pretreated with peiminine for 2 h. GST-rRANKL (50 ng/ml) was then added to each well, and total protein was harvested in RIPA buffer at the following time points: 0, 10, 20, 30, and 60 min.

The proteins were separated by sodium dodecyl sulfate-polyacrylamide gel electrophoresis (10%), transferred onto nitrocellulose membranes, and blocked with 5% BSA for 2 h. The membranes were incubated with the following primary antibodies: anti-c-Fos (1:1,000, Cat. #2250), anti-NF-κB (anti-p65) (1:1,000, Cat. #8242), anti-phospho-NF-κB (anti-pp65) (1:1,000, Cat. #3033), anti-NFATc1 (1:1,000, Cat. #sc-7294), anti-integrin β3 (1:1,000, Cat. #sc-365679), anti-cathepsin K (CTSK) (1:1,000, Cat. #sc-48353), anti-ATP6v0s2 (1:1,000, Cat. #sc-69108), anti-p-IκB-α (1:1,000, Cat. #sc-8404), and anti-β-actin (1:5,000, Cat. #sc-47778), anti-phospho-ERK1/2 (1:1,000, Cat. #AF1015), anti-ERK1/2 (1:1,000, Cat. #AF0155), anti-phospho-P38 (1:1,000, Cat. #AF4001), anti-P38 (1:1,000, Cat. #AF6456).

Primary antibodies specific for c-Fos, phospho-NF-κB (pp65), and NF-κB (p65) were acquired from Cell Signaling Technology (Beverly, MA, USA). Primary antibodies specific for NFATc1, integrin β3, CTSK, V-ATPase-d2 (ATP6v0d2), phosphorylated iκB (p-IκB)-α, and β-actin were obtained from Santa Cruz Biotechnology (San Jose, CA, USA). Primary antibodies specific for ERK1/2, phospho-ERK1/2, phospho-P38, and P38 were acquired from Affinity Biosciences (Jiangsu, China).

The membranes were incubated with primary antibodies at 4°C for more than 12 h and then with secondary antibodies conjugated to horseradish peroxidase at room temperature for more than 2 h. Immunoreactive bands were visualized with the Touch Imaging System made by Bio-Rad (ChemiDoc™, Bio-Rad, CA, USA).

The relative protein content was calculated with ImageJ software as follows: adjust the protein band images to 8-bit and obtain clean protein bands using the *Subtract Background* function; use the *Measurement* function to calculate the gray value of each band; divide the gray values of the control proteins by the gray values of the corresponding target proteins to determine the relative protein amounts.

### Ovariectomy Mouse Model

Female 10-week-old C57BL/6 mice (n=30), acquired from the Animal Experimental Center of Sir Run Run Shaw Hospital (Zhejiang, China), were randomly separated into three groups: the sham group (control group) (n=10), the OVX group (n=10), and the intervention group (n=10). Mice from the OVX and intervention groups were ovariectomized according to the method published by Zhou in 2016 (27). As a control, mice in the sham group underwent a sham operation; mice in the intervention group were treated with peiminine (10 mg/kg), while those in the OVX and sham groups were injected with PBS.

All mice were housed in ventilated and sterilized cages and subjected to surgery after adaptive feeding for 1 week. Each of these specific pathogen-free cages held five mice. Seven days after the surgery, mice in the OVX and sham groups were administered PBS, while those in the intervention group were intraperitoneally injected with peiminine (10 mg/kg) every 2 days for 6 weeks. All the mice survived and were healthy in the interim. At the seventh week after surgery, all the mice were sacrificed, and their femurs were harvested for histological and micro-CT (μCT) analysis.

### µCT Scanning of Mouse Femurs

We measured the following parameters of mouse femur samples: the bone volume/tissue volume ratio (BV/TV), trabecular number (Tb. N), trabecular separation (Tb. Sp), and trabecular thickness (Tb. Th). Samples were scanned with a BRUKER skyscan1176 μCT instrument (Bruker Daltonic Inc. USA) after fixation with 4% paraformaldehyde for 1 day (24 h). The μCT setup was as follows: 50 kV scanning voltage, 500 μA scanning current, 9 μm spatial resolution, and 1,600 × 2,672-pixel image matrix. Then, a 1-mm-high area 0.5 mm beneath the growth plate was designated for qualitative and quantitative analysis. N-Recon software was used for three-dimensional image rebuilding, and CT-AN software was used for quantitative analysis.

### Histological Staining

Left femur samples from all mice were fixed in 4% paraformaldehyde for 2 days (48 h) and then decalcified by 14% EDTA at 37°C for 14 days (9). Sagittal paraffin sections at thickness of 5 μm were obtained from the decalcified bone tissues. Representative images of TRAP staining and hematoxylin and eosin (H&E) staining were collected, and the claret red cells around the resorbed bone were considered TRAP-positive OCs. In addition, we assessed the OCs surface (OC. S) and bone surface (BS) by measuring the TRAP-positive cells’ perimeters and bone perimeters separately.

### Statistical Analysis

In the *Results* section, all quantitative data are presented as the mean ± standard deviation. Statistical analyses were conducted using one-way analysis of variance (ANOVA) followed by Tukey’s *post hoc* test with GraphPad Prism 8. All data are presented as the means ± SDs; *P < 0.05, **P < 0.01, ***P < 0.001 compared to the control.

## Results

### Peiminine Is Not Toxic in BMMs and Suppresses OC Formation and Fusion Induced by RANKL

Before assessing the inhibitory effect of peiminine on osteoclastogenesis, we investigated its cytotoxicity. The CCK-8 assays confirmed that the reduction in the number of OCs was not due to BMM cytotoxicity, as the number of live cells did not differ after treatment with varying concentrations of peiminine as determined by absorbance detection (**Figure 1B**). The number of cells in each group did not change significantly in the presence of various concentrations of peiminine, indicating that peiminine had no toxic effect on living BMMs (1 μmol/L group: p= 0.0665; 1 μmol/L: p= 0.0503; 5 μmol/L: p= 0.0909; 10 μmol/L: p=0.0682; 20 μmol/L: p=0.3824; 40 μmol/L: p=0.3174).

To evaluate the inhibitory effect of peiminine on the generation of OCs, TRAP staining was used to assess the cellular responses to increasing peiminine concentrations (1, 2.5, 5, and 10 μmol/L). As shown in **Figures 1C, D**, the number of TRAP-positive OCs in each well was dose-dependently decreased, and significantly fewer multinuclear TRAP-positive cells were observed in the cells treated with 10 μmol/L peiminine than in untreated cells (p= 2.04798E-09). Similarly, the numbers of cells treated with 1, 2.5, and 5 mol/L peiminine were obviously decreased (1 μmol/L: p= 0.0129; 2.5 μmol/L: p= 6.93569E-06; 5 μmol/L: p= 1.27028E-08).

Additionally, to determine whether peiminine disrupts the cellular fusion of OCs, fluorescence staining was performed to assess their fusion and the quantity of nuclei in every multinucleated cell. **Figures 1E, F** show that cell fusion was reduced in the presence of 5 and 10 μmol/L peiminine, which is consistent with the TRAP staining results. Specifically, the number of nuclei per OC was markedly decreased (5 μmol/L: p= 0.0004652; 10 μmol/L: p= 9.86236E-05).

### The Inhibitory Effect of Peiminine Is Time-Dependent

**Figure 2:**
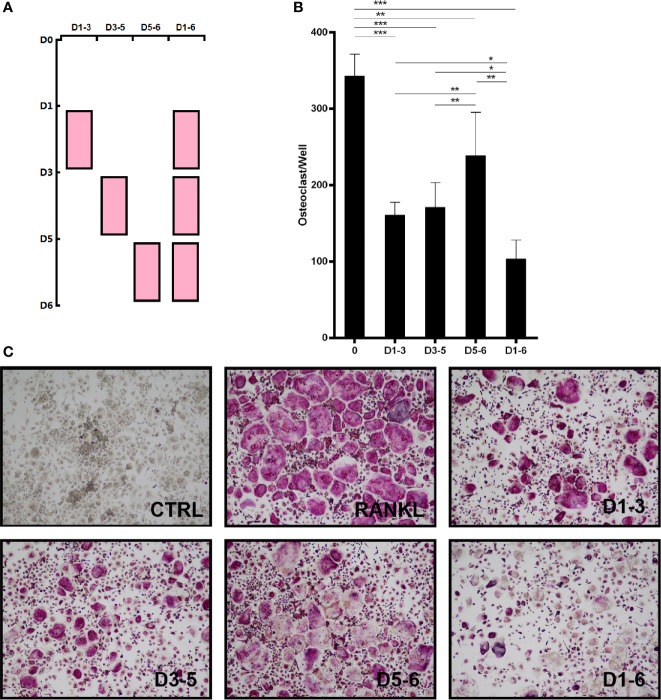
Peiminine has a suppressive effect on OC formation at different time points. **(A)** BMMs were treated with peiminine on days 1–3, 3–5, 5–6, and 1–6. **(B)** Quantitative analysis of the OC numbers in groups with different administration times (TRAP-positive cells were counted as OCs). n= 5, *P < 0.05, **P < 0.01, ***P < 0.001. **(C)** Representative images of the TRAP-positive cells in the different administration time groups.

In addition, BMMs were treated with peiminine on days 1–3, 3–5, 5–6, and 1–6 to assess its inhibitory effect at various stages of OC differentiation. In **Figure 2A**, the pink boxes represent the time points of the dosing. Compared with the control group, peiminine had a notable inhibitory effect in all treatment groups. The number of OCs in the 1–6day group was dramatically lower than that in the control group (p=0.00000060), and those in the remaining groups were lower than that in the control group (1–3day group: p= 0.00000175; 3–5day group: p= 0.0000189; 5–6day group: p= 0.00636).

Moreover, direct comparisons of the treatment groups revealed that the effect of peiminine was limited on days 5–6, as the number of OCs in the 5–6day group was higher than that in the other treatment groups (1–3day group: p= 0.019; 3–5day group: p= 0.049; 1–6day group: p= 0.001). Additionally, peiminine exerted an optimal inhibitory effect on the 1–6day group, as the OC number was lower than that in the other groups (1–3 group: p= 0.002; 3–5 group: p= 0.005; 5–6 group: p= 0.001) (**Figures 2B, C**).

### Peiminine Suppresses the Resorptive Activity of OCs

To further demonstrate the ability of peiminine to disrupt OC resorption, BMMs were seeded on bone slices and treated with or without varying concentrations of peiminine (5 and 10 μmol/L) together with M-CSF (50 ng/ml) and GST-rRANKL (50 ng/ml). Then, the freeze-dried bone slices were observed by SEM, and the resorption area was measured with ImageJ.

**Figure 3:**
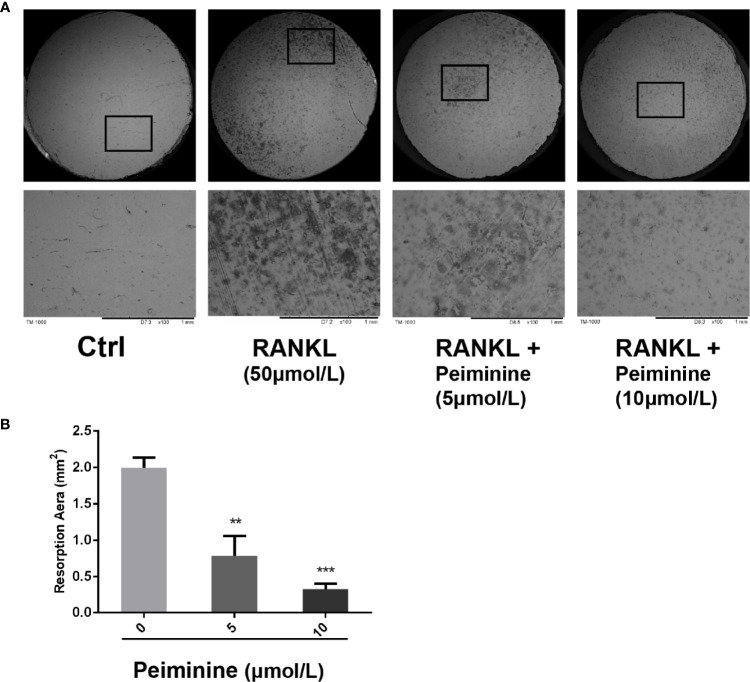
OC resorption is inhibited by peiminine. **(A)** Representative images of the bone slice resorption areas in the different groups. BMMs in the first group (control) were neither induced by RANKL nor treated with peiminine; BMMs in the second group were induced by RANKL but not treated with peiminine; and BMMs in the third and fourth groups were induced by RANKL and treated with peiminine at concentrations of 5 µmol/L and 10 µmol/L, respectively. **(B)** The bone slice resorption areas in all groups were quantitatively analyzed. n= 3, **P < 0.01, ***P < 0.001.

Compared to the control, the non-treatment group, induced by GST-rRANKL, was expected to have the maximum number of OCs and to exhibit the most obvious effect on resorption. Owing to the inhibitory effect of peiminine on OC function, the bone slices in the intervention group were expected to have a smaller resorption area. According to the SEM results and analysis of the data shown in **Figures 3A, B**, the peiminine-free group had the largest resorption pit area, and the resorption area decreased as the drug concentration increased (5 μmol/L: p= 0.0071; 10 μmol/L: p= 0.0005). The results obtained from bone slices were consistent with our hypothesis.

### Peiminine Downregulates Formation- and Function-Related Genes

**Figure 4:**
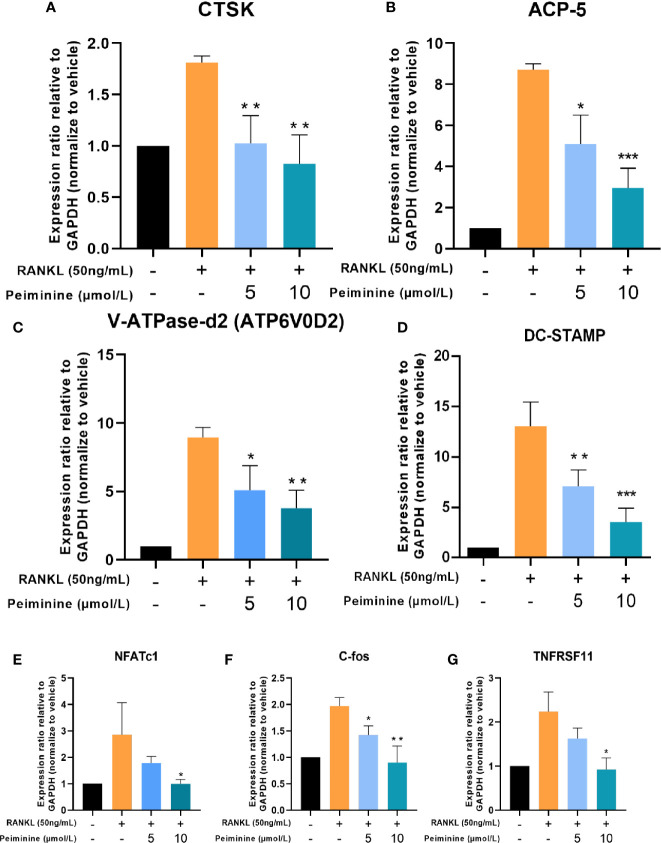
Osteoclastogenesis-related genes are downregulated by peiminine. **(A)** CTSK, **(B)** ACP-5, **(C)** V-ATPase-d2 (ATP6V0D2), **(D)** DC-STAMP, **(E)** NFATc1, **(F)** c-Fos, and **(G)** TNFRSF11. GAPDH was selected as the control gene. n= 3, *P < 0.05, **P < 0.01, ***P < 0.001.

The expression levels of genes critical for RANKL-induced OC differentiation and function, including NFATc1, c-Fos, Acp5 (TRAP), ATP6V0D2, DC-STAMP, and TNF, were analyzed by qRT-PCR to further investigate the mechanism underlying the inhibitory effect of peiminine. Compared with those in the control group, the expression levels of OC-related genes in the RANKL-treated group were substantially elevated. The expression levels of genes related to OC function, such as CTSK (5 μmol/L: p= 0.0082; 10 μmol/L: p= 0.0040) and Acp5 (5 μmol/L: p= 0.0120; 10 μmol/L: p= 0.0005) (**Figures 4A, B**), and fusion-related genes, such as ATP6v0d2 (5 μmol/L: p= 0.0263; 10 μmol/L: p= 0.0039) and DC-STAMP (5 μmol/L: p= 0.0246; 10 μmol/L: p= 0.0041) (**Figures 4C, D**), were detected in cells treated with peiminine at concentrations of 5 and 10 μmol/L.

Similarly, after peiminine intervention, genes related to OC formation (NFATc1 and c-Fos) were downregulated (NFATc1: 5 μmol/L: p= 0.2051; 10 μmol/L: p= 0.451; c-Fos: 5 μmol/L: p= 0.0158; 10 μmol/L: p= 0.0061) (**Figures 4E, F**). In addition, the expression of the TNFRSF11 gene, which encodes the RANK protein, was inhibited by peiminine (5 μmol/L: p= 0.1065; 10 μmol/L: p= 0.0119) (**Figure 4G**). These results confirm that peiminine indeed inhibited the RANKL-induced differentiation and function of OCs.

### Peiminine Suppresses the Expression of Critical Proteins of RANKL-Induced OCs

**Figure 5:**
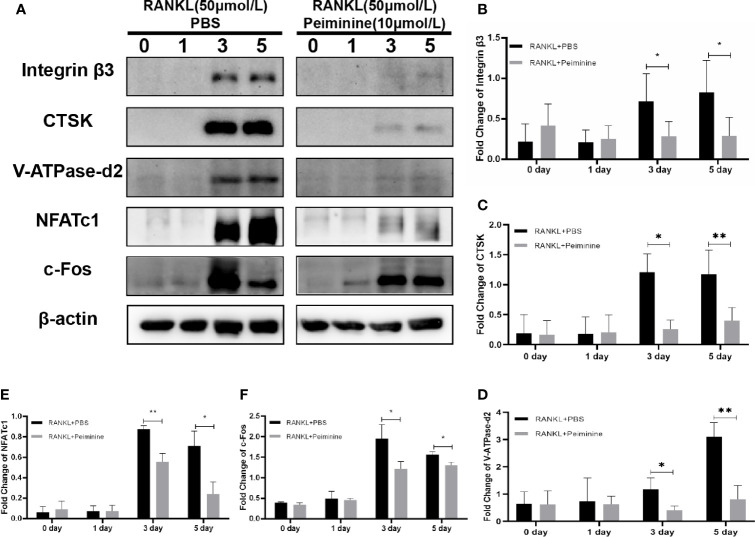
c-Fos, NFATc1 and downstream proteins are suppressed by peiminine. **(A)** Western blots analysis of the integrin β3, CTSK, V-ATPase-d2, NFATc1, and c-Fos expression induced by treatment with RANKL (50 ng/mL) for 0, 1, 3, and 5 days in the presence or absence of peiminine (10 μmol/L). Quantitative analysis of the differential expression levels of **(B)** integrin β3 (n= 5), **(C)** CTSK (n= 3), **(D)** V-ATPase-d2 (n= 3), **(E)** NFATc1 (n= 3), and **(F)** c-Fos (n= 3). The β-actin was selected as the control protein. *P < 0.05, **P < 0.01.

The Western blot results were consistent with the qRT-PCR results, demonstrating that peiminine effectively inhibited the expression of essential genes and proteins related to OC differentiation and thereby reduced the expression of downstream genes and proteins associated with OC function. The expression levels of a series of critical factors in the control and experimental groups were measured by Western blot, and the results are shown in **Figure 5A**.

As mentioned previously, integrin β3, CTSK, and ATP6v0d2 are critical for OC function, and their protein expression levels were reduced in cells treated with peiminine (integrin β3: 0-day: p= 0.9161; 1-day: p= 0.6606; 3-day: p= 0.0373; 5-day: p= 0.0306; CTSK: 0-day: p= 0.8770; 1-day: p= 0.9627; 3-day: p= 0.0167; 5-day: p= 0.0028; ATP6v0d2: 0-day: p= 0.9432; 1-day: p= 0.8401; 3-day: p= 0.0440; 5-day: p= 0.0055) (**Figures 5B–D**).

NFATc1 and c-Fos, regulatory factors upstream of the abovementioned proteins, were also remarkably downregulated by peiminine in the 3- and 5-day groups (NFATc1: 0-day: p= 0.6205; 1-day: p= 0.9749; 3-day: p= 0.0031; 5-day: p= 0.0117; c-Fos: 0-day: p= 0.1781; 1-day: p= 0.7359; 3-day: p= 0.0296; 5-day: p= 0.0152) (**Figures 5E, F**).

### The NF-κB and ERK1/2 Signaling Pathways Are the Potential Targets of Peiminine

**Figure 6:**
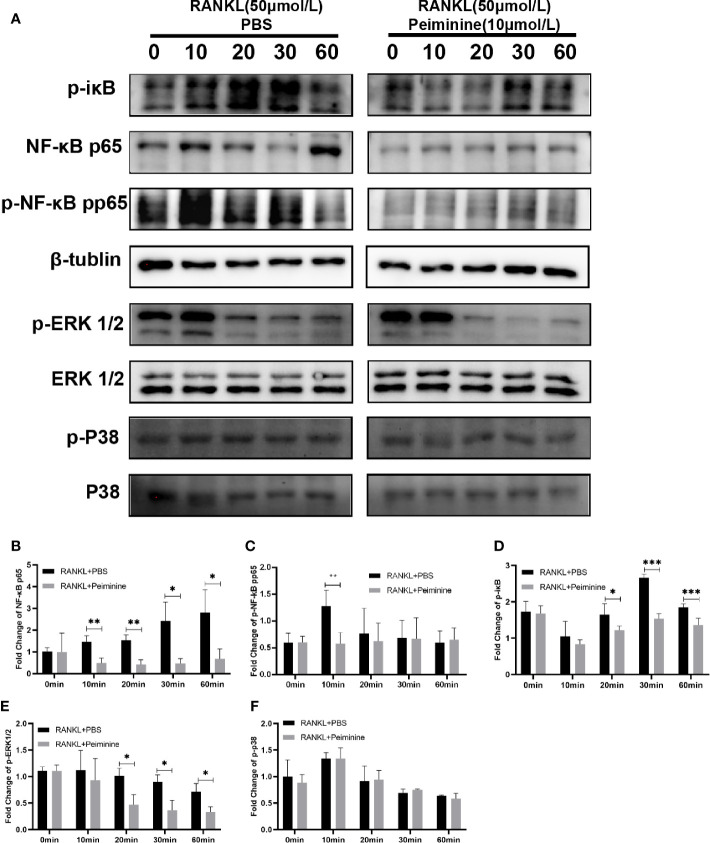
Peiminine interferes with the NF-κB and ERK1/2 pathways. **(A)** Western blot analysis of NF-κB, p-iκB, p-NF-κB, p-ERK1/2, and p-P38 in cells treated with RANKL (50 ng/mL) for different amounts of time (0, 10, 20, 30, and 60 min) in the presence or absence of peiminine (10 μmol/L). Quantitative analysis of the differential expression levels of **(B)** NF-κB (n= 3), **(C)** p-NF-κB (n= 4), **(D)** p-iκB (n= 4), **(E)** p-ERK1/2 (n= 3), **(F)** p-P38 (n= 3). β-tublin was selected as the control protein for the normalization of p-iκB, NF-κB, p-NF-κB, and the ERK1/2 for p-ERK1/2, the P38 for p-P38. *P < 0.05, **P < 0.01, ***P < 0.001.

A key molecular event of early RANKL-induced osteoclastogenesis is the activation of NF-κB (28, 29), and activated NF-κB is an integral upstream regulator of NFATc1 (30). As shown in **Figure 6B**, the levels of NF-κB were notably downregulated by peiminine in the presence of RANKL for 10 min (p= 0.0097), 20 min (p= 0.0043), 30 min (p= 0.0195), and 60 min (p= 0.0324). As shown in **Figure 6C**, the level of Phosphorylated NF-κB (p-NF-κB) was also decreased by peiminine (p= 0.0083).

As an important upstream regulator, iκB can prevent the entry of NF-κB into the nucleus; however, during the process of OC differentiation, iκB is phosphorylated by the combination of RANK and RANKL (31). **Figure 6D** shows that peiminine obviously reduced the expression of p-iκB at 20 min (p= 0.0388), 30 min (p= 1.42687E-05), and 60 min (p= 0.0031) and consequently diminished the levels of p-NF-κB and NF-κB. These findings suggested that peiminine inhibited the NF-κB pathway and thus suppressed OC differentiation and function.

Moreover, MAPK signaling pathways are important regulators of downstream activation (16, 32). We found that the levels of p-ERK1/2 were decreased in cells treated with peiminine for 20 min (p= 0.0166), 30 min (p= 0.0154), and 60 min (p= 0.0208). However, peiminine had no significant effect on the p-P38 level (**Figures 6E, F**).

### Peiminine Plays a Role of Alleviation Against Bone Loss *In Vivo*

**Figure 7:**
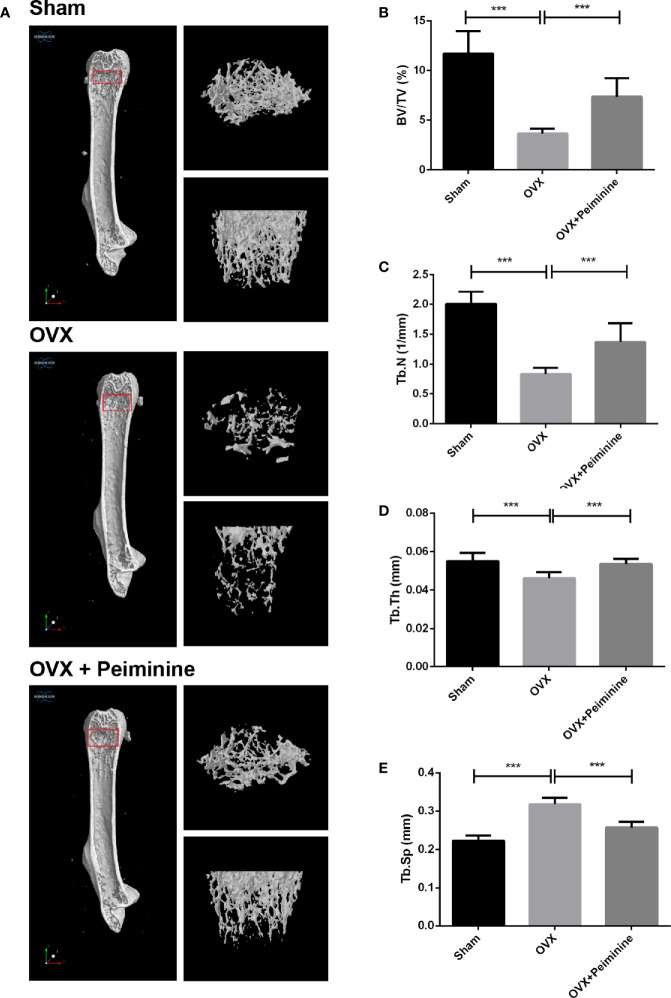
Peiminine ameliorates the systematic bone loss induced by OVX. **(A)** Computer-generated 3D high-resolution micro-CT image of the femur microstructure. Quantitative measurements of **(B)** BV/TV, **(C)** Tb. N, **(D)** Tb. Th, and **(E)** Tb. Sp in the control group (sham), non-treatment group (OVX), and treatment group (OVX+peiminine). n= 10, ***P < 0.001.

Animal experiments were performed to evaluate the bio-effect of peiminine *in vivo*. μCT and histomorphometric analyses showed that peiminine obviously alleviated the resorption of bone caused by OCs (**Figure 7A**). The BV/TV was obviously higher in the peiminine-treated group than in the OVX group (p= 1.03253E-05) (**Figure 7B**). Similarly, the Tb. N and Tb. Th were higher in the peiminine-treated group than in the OVX group (Tb.N: p= 6.72736E-05; Tb.Th: p= 2.21584E-05) (**Figures 7C, D**), while the Tb. Sp in the intervention group was lower than that in OVX group (p= 1.55671E-07) (**Figure 7E**).

**Figure 8:**
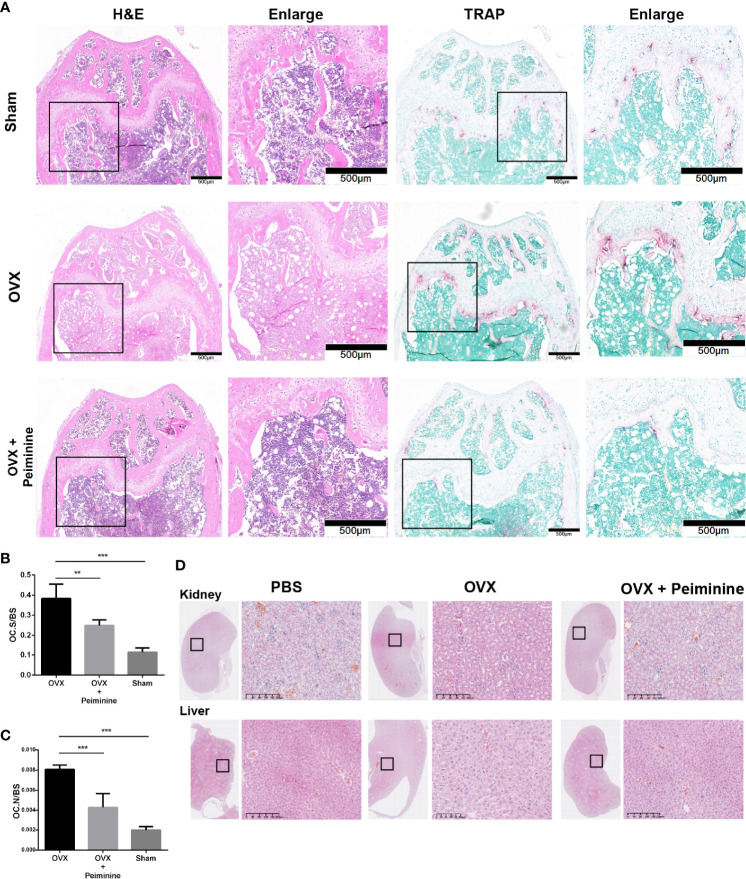
Peiminine had no bio-toxicity and ameliorated the bone loss induced by OVX by inhibiting osteoclastogenesis in vivo. **(A)** Representative H&E and TRAP staining images of decalcified bones from mice in the sham, OVX, and peiminine (10 mg/kg) treatment groups. **(B)** Representative H&E staining images of kidneys and livers of mice from the control and experimental groups. Quantitative measurements of **(B)** OC. S/BS; **(C)** OC. N/BS, in the control group (Sham), non-treatment group (OVX), and treatment group (OVX + peiminine). n= 5, **P < 0.01, ***P < 0.001. **(D)** Representative H&E staining images of kidneys and livers of mouse from control and experimental groups.

The histomorphometric analysis results further supported the findings presented above. H&E staining indicated the distribution of OCs and trabecular bone *in vivo*, and the result was consistent with that obtained by μCT analysis. Then, OCs at corresponding positions were stained, revealing that sections from peiminine-treated OVX mice had significantly fewer TRAP-positive cells than those from untreated OVX mice (**Figure 8A**). The OC. S/BS (p= 0.004493691) and OC. N/BS (p= 0.000363396) results also indicated that peiminine inhibited osteoclastogenesis in bone tissue (**Figures 8B, C**).

Additionally, H&E staining of liver and kidney tissues harvested from the mice showed no lesions in any of the groups (**Figure 8D**). The lack of peiminine toxicity *in vivo* corresponded with the results of the CCK-8 assay described in the section *Peiminine Is Not Toxic in BMMs and Suppresses OC Formation and Fusion Induced by RANKL*.

## Discussion

In this study, TRAP staining was used as the major determination method to demonstrate many indices of osteoclastogenesis, because TRAP is associated with oxidative stress and a crucial indicator of OCs (33). According to our results, under the premise of peiminine’s biosafety, we verified peiminine’s inhibitory effect in different concentrations and stages. Furthermore, the fluorescence staining and bone slice resorption results strengthen the TRAP staining results and together show that peiminine suppressed both OC fusion and function.

The following experiments were carried out to confirm the conclusions above at the molecular level. The role of NFATc1 is critical throughout osteoclastogenesis (34), and according to previous research, c-Fos is the indispensable bridge between RANK and NFATc1 (17, 35, 36). As shown in **Figures 4** and **5**, the levels of the gene TNFRSF11, which encodes for RANK, and the genes NFATc1 and c-Fos were in decline within the groups treated with peiminine. Results above suggest that peiminine might affect the whole RANK-NFATc1 pathway. Therefore, it can be speculated that peiminine decreases the levels of downstream factors, including CTSK, and Integrinβ3, which are crucial for OCs’ resorption (37–39); as well as DC-STAMP and ATP6v0d2, which are indispensable in OCs’ cellular fusion and maturation (3, 40–43).

The activation of NF-κB is an important event that occurs in the early stage of RANKL-induced OC differentiation and is vital for the activation of NFATc1 (44). NF-κB is mainly present in the cytosol in the form of a dimer consisting of iκB and NF-κB, and iκB can prevent NF-κB from entering the nuclei (17, 45). RANKL can induce the phosphorylation of iκB and lead to the degradation of iκB. NF-κB is activated in the absence of iκB, and p-NF-κB enters the nucleus and is involved in the activation of NFATc1 (32). As shown in **Figures 6A, C, D**, peiminine downregulated the expression of NF-κB and p-NF-κB, suggesting that it has an inhibitory effect on NFATc1. We also found that peiminine significantly decreased the level of p-iκB, suggesting that it suppresses the degradation of iκB by inhibiting iκB phosphorylation. This phenomenon explains why the levels of NF-κB and p-NF-κB were reduced in the presence of peiminine.

**Figure 9:**
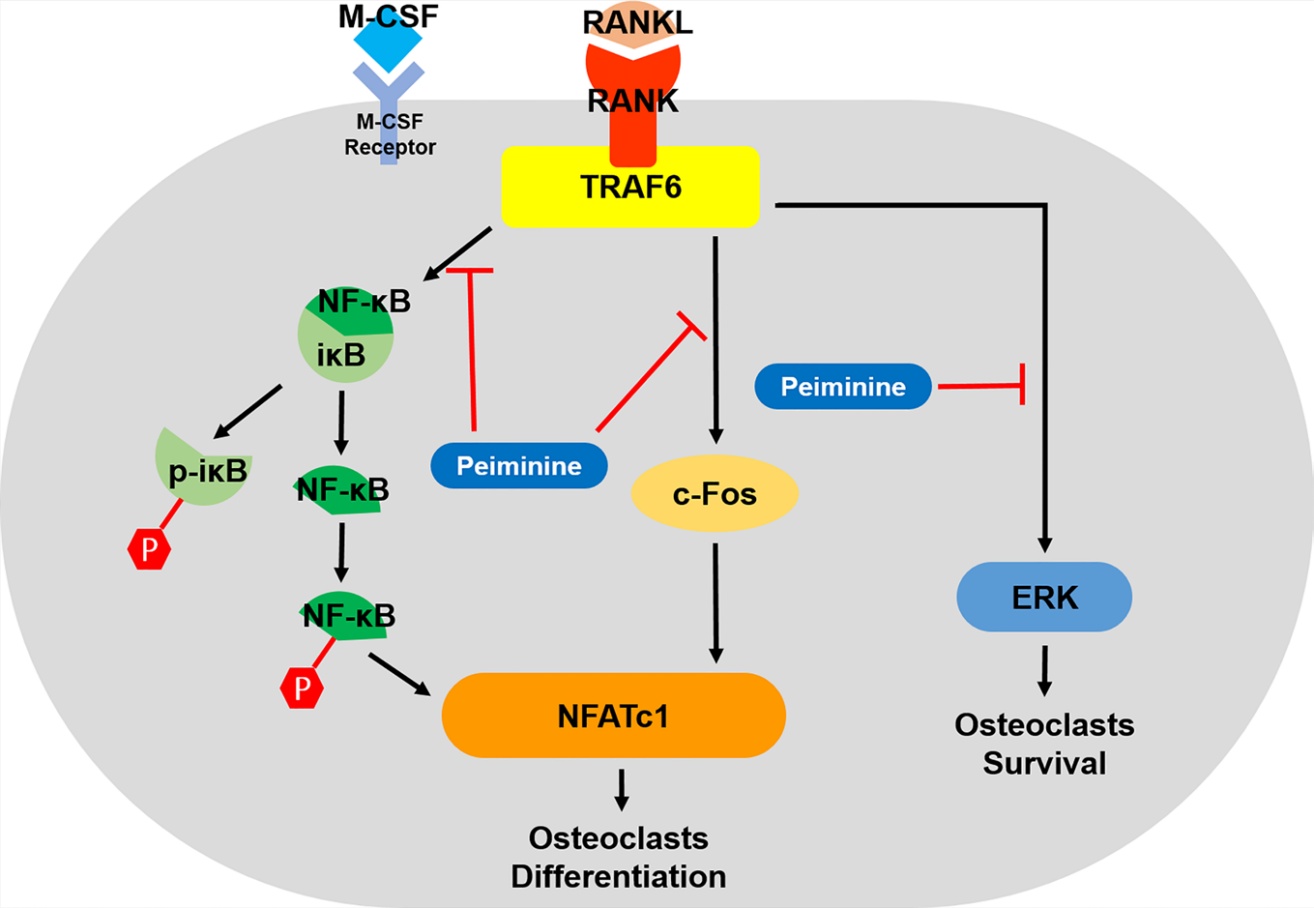
Schematic diagram of how peiminine suppresses RANKL-induced osteoclastogenesis.

MAPK pathways, including ERK, JNK, and P38, are associated with OC formation and function (36, 46), and ERK is indispensable for OC survival (18, 35). We therefore assessed these two signaling pathways, revealing that peiminine downregulated ERK1/2 in the early stage of osteoclastogenesis but did not affect the expression of p-P38. Peiminine inhibited osteoclastogenesis and obviously prevented bone loss in OVX mice, most likely by interfering with the NF-κB, ERK and c-Fos-NFATc1 pathways (**Figure 9**).

Estrogen has the ability to block osteoclastogenesis and increase OC apoptosis, and the withdrawal of estrogen increases the number of OCs and promotes bone resorption (47). Thus, we used OVX mouse as the experimental animal model. Animal experiments further supported the results *in vitro*. Because peiminine can presumably alleviate estrogen deficiency-induced osteoclastogenesis *in vivo*. The μCT and histological analysis results suggested that peiminine effectively decreased the level of osteoporosis in OVX mice, as relatively mild bone loss was observed in these mice treated with peiminine. Besides, the biosafety of peiminine was reconfirmed by animal experiments, as none of the mice injected with peiminine exhibited an abnormal reaction or hypersensitivity, demonstrating its safety.

In this study, we assessed the alleviating effect of peiminine *in vivo* after systemic administration, but there is room for improvement. For example, whether the circumstances of systemic peiminine administration affect bone homeostasis or cause side effects remains unknown. The inclusion of an extra sham-operated group of mice treated with peiminine could help to assess the effect of the drug in the steady state. Additionally, the serum detection of bone turnover markers, such as CTX, OPG, P1NP, and RANKL, after peiminine treatment would presumably further elucidate its mechanism of action.

In addition, the mode of peiminine needs to be further investigated. In this study, OVX mice were treated before the induction of osteoporosis, which indicated the alleviating effect of peiminine on bone loss, but whether peiminine can ameliorate previous bone loss remains to be determined. In the future, we plan to administer peiminine to osteoporotic mice to study its therapeutic effect upon osteoporosis. It is also important to explore the effects of the drug on mice undergoing long-term treatment, as these experiments can provide more information on the long-term impacts of peiminine on bone quality and potential side effects and thereby aid the development of more scientific therapeutic strategies.

In the future, we hope to study additional dosing strategies. A newly developed smart nanosacrificial layer was shown to precisely target and inhibit osteoclast function. Nanomaterials modified by tetracycline with bone-targeting properties can be used to encapsulate intervening drugs, thereby yielding better targeted drug delivery than that achieved *via* systemic administration (48). This represents a promising research direction for the application of peiminine in the future.

## Conclusion

This study demonstrates that peiminine, a natural herb-extracted compound, can inhibit the formation and function of OCs *via* multiple targets and is therefore a promising novel therapeutic agent for osteoporosis.

## Data Availability Statement

The original contributions presented in the study are included in the article/supplementary material. Further inquiries can be directed to the corresponding authors.

## Ethics Statement

The animal study was reviewed and approved by Laboratory Animal Center of Sir Run Run Shaw Affiliated Hospital of Zhejiang University School of Medicine.

## Author Contributions

MZ and SW contributed to conception. MZ, LS, and TW designed the study, and MZ wrote the first draft of the manuscript. WX, JZ, and YW organized the database. YG, RY, and YC performed the statistical analysis. BZ, ZW, and JZ wrote sections of the manuscript. All authors contributed to the article and approved the submitted version.

## Funding

This project was supported by the National Natural Science Foundation of China (Grant Nos. 81572207, 81201435, 82101647), Natural Science Fund of Zhejiang Province (LQ20H060005) Medical Health Science and Technology Project of Zhejiang Provincial Health Commission (2021436226), Health Commission of Shanxi Province (Grant No. 2020075), and the Public Projects of Zhejiang Province (LGF19H060013).

## Conflict of Interest

The authors declare that the research was conducted in the absence of any commercial or financial relationships that could be construed as a potential conflict of interest.

## Publisher’s Note

All claims expressed in this article are solely those of the authors and do not necessarily represent those of their affiliated organizations, or those of the publisher, the editors and the reviewers. Any product that may be evaluated in this article, or claim that may be made by its manufacturer, is not guaranteed or endorsed by the publisher.
